# PCE simulation toolkit: a platform for perceptual crossing experiment research

**DOI:** 10.3389/fnbot.2023.1048817

**Published:** 2023-05-17

**Authors:** Federico Sangati, Rui Fukushima

**Affiliations:** ^1^Cognitive Neurorobotics Research Unit, Okinawa Institute of Science and Technology, Okinawa, Japan; ^2^Embodied Cognitive Science Unit, Okinawa Institute of Science and Technology, Okinawa, Japan

**Keywords:** perceptual crossing experiment, evolutionary algorithm, CTRNN, simulation tool for artificial life, visualization tool for artificial life

## Abstract

The Perceptual Crossing Experiment (PCE) has been the object of study for over a decade, and aims at explaining how we perceive, interact with, and understand each other in real-time. In addition to human participant studies, a number of computational models have investigated how virtual agents can solve this task. However, the set of implementation choices that has been explored to date is rather limited, and the large number of variables that can be used make it very difficult to replicate the results. The main objective of this paper is to describe the PCE Simulation Toolkit we have developed and published as an open-source repository on GitHub. We hope that this effort will help make future PCE simulation results reproducible and advance research in the understanding of possible behaviors in this experimental paradigm. At the end of this paper, we present two case studies of evolved agents that demonstrate how parameter choices affect the simulations.

## 1. Introduction

The Perceptual Crossing Experiment (PCE), has been studied for over a decade, and aims at explaining how we perceive, interact with, and understand each other in real-time. The experiment was inspired by the double-video projection study of Murray and Trevarthen (1985), in which infants were observed when interacting with a video showing their mothers in real-time vs. a prerecording from a previous interaction. The fact that infants responded differently to these two conditions (being distressed in the playback situation) suggests that dynamics of real-time interaction and social contingency constitute an intrinsic property of social perception. Although, the mechanisms of this process are yet to be fully understood.

Auvray et al. (2009) first introduced PCE as a minimal experimental version of the double-video projection study via the real-time interaction between two human participants. A number of studies have followed, attempting to replicate the experiment with human subjects and extending it further (Lenay et al., 2010; Auvray and Rohde, 2012; Lenay, 2012; Froese et al., 2014; Iizuka et al., 2015). Most of the studies were conducted on adult healthy individuals, although it has also been proposed as a method for testing real-time dyadic embodied interaction in the context of mental pathology such as schizophrenia (Zapata-Fonseca et al., 2021). At the same time, there has also been a growing interest in computational modeling to replicate this experiment using evolutionary robotics algorithm (Rohde and Di Paolo, 2006; Iizuka and Di Paolo, 2007; Di Paolo et al., 2008; Froese and Di Paolo, 2008; Froese and Di Paolo, 2010; Froese and Di Paolo, 2011; Izquierdo et al., 2022). The objective of these studies was to gain additional insights into the dynamics of the minimal social interaction process. Unfortunately, all these computational models make use of very specific implementation choices, which have been proven hard to replicate (Izquierdo et al., 2022), as no source code has been made available to the current date. This might constitute a serious obstacle to the development of consistent research on PCE simulations and underlying cognitive mechanisms.

The main contribution of the current work is providing an open-source implementation of simulation-based PCE, in the hope of facilitating the process of reproducing and extending simulation results. The code is available on GitHub^1^ as a Python library which incorporates a set of visualizations tools, such as plots, network diagrams, and visual animations of the agent's behavior, which can serve to gather better insight into the dynamics of the agents when attempting to solve the task. The implementation makes use of vectorized operations using the NumPy library, and multi-core optimization, for improving performance.^2^ The code provides a large number of parameters that can be changed through appropriate arguments while preserving enough flexibility to be extended for new purposes. We invite other researchers interested in using and extending this library to consult the README file which explains its functionality in more technical detail.

The rest of the paper is organized as follows. Firstly we will briefly introduce the Perceptual Crossing Experiment and previous simulations using this paradigm. In Section 2, we describe the PCE Simulation Toolkit we have implemented and detail the implementation behind the simulated agents. Section 3 presents two case studies of evolved agents that highlight some assumptions made in previous studies. Finally, in Section 4, we conclude and discuss future work.

### 1.1. Perceptual crossing experiment

**Figure 1:**
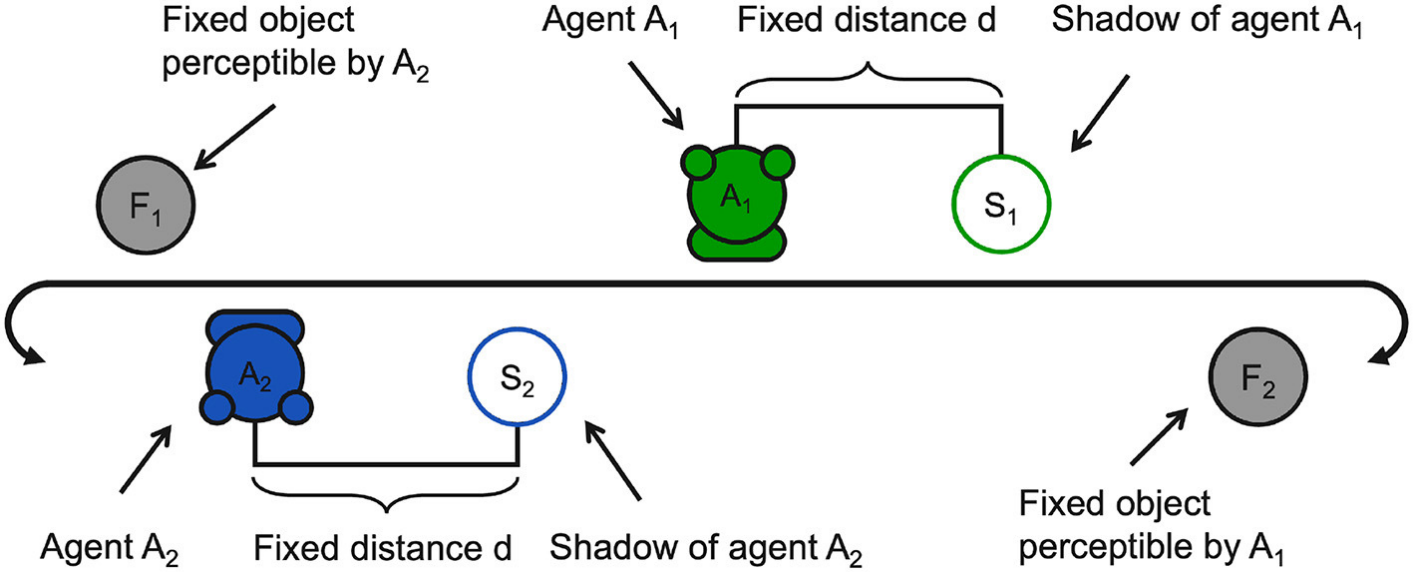
A schematic illustration of the PCE environment.

**Figure 2:**
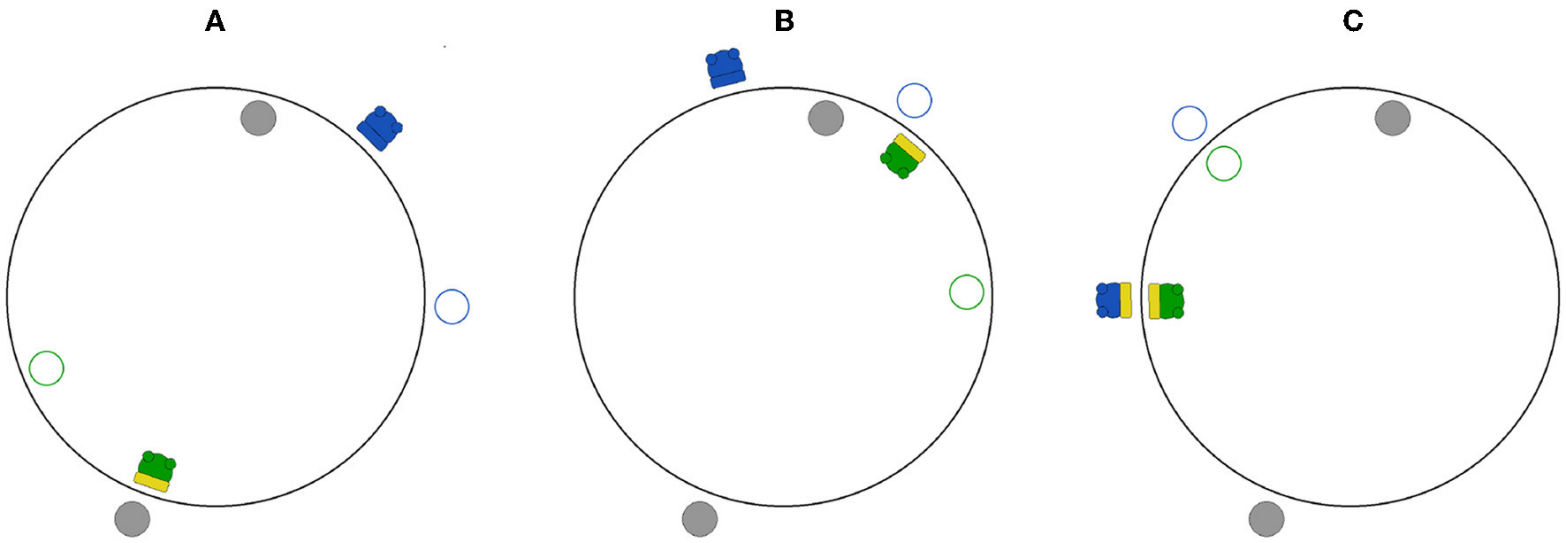
The PCE simulation environment as rendered by our visualization tool for 3 different time steps in a sample simulation. Gray-filled circles represent static objects. Empty circles with a colored border (green or blue) indicate the shadows of the respective agent with the same color. Agents are represented by filled colored circles (green or blue) with wheels, and a rectangular sensor facing outwards or inwards (depending on the agent's position). The sensor is depicted in yellow when the agent senses the presence of the fixed object on the opposite side **(A)**, the shadow of the other agent **(B)**, or the other agent **(C)**.

In the perceptual crossing experiment, two agents coexist in a one-dimensional environment, as shown in the diagram in Figure 1. The environment wraps around forming a ring as shown in Figure 2. The agents are placed in the environment facing each other and can freely move around the ring in either direction. By means of a binary sensor, covering the whole width of the agent, they are able to *sense* the presence of a number of elements when crossing them:^3^ (i) a static object facing the agent, (ii) the other agent, and (iii) the shadow of the other agent which is positioned at a fixed distance with respect to the other agent. The agents are free to move past all perceived elements unimpeded (without collision or friction).

The agents are expected to interact in the environment and learn to identify each other from among other objects that can be encountered (the shadow replicating the movement of the other agent, and the fixed object). In the original experiment with human subjects, this objective was operationalized by asking each participant to press a button whenever they believed to be crossing the other subject, and assessing how accurate their response was. In the simulation models, this objective has been simplified by assessing how long agents are able to stay close together.

### 1.2. Previous PCE simulation work

It is beyond the scope of this paper to provide a detailed summary of previous simulation models. However, we would like to discuss some implementation choices that apply to most.

Firstly, in all proposed models, the evolved agents are tested against *clones* of themselves. The only exception is the work of Iizuka and Di Paolo (2007), however their model is a very simplistic version of PCE without fixed objects and shadows. We believe the choice of using clones is a very restricted (albeit interesting) case to be studied because, in the human-subject experiments, the two agents are controlled by different individuals who may adopt different strategies. Froese and Di Paolo (2008) justifies this choice by referring to the work by Iizuka and Ikegami (2004), which suggests that genetically similar agents are potentially better at coordination. This fails at capturing solutions where the two agents may develop complementary strategies, as we will show in Case Study II of Section 3.2. An alternative justification (Froese and Di Paolo, 2011) is related to the claim that agents can have an identical architecture, but they can still reach different types of strategies because the different interaction histories they go through may lead to different state dynamics. We are not aware of any previous work explicitly reporting such a scenario and showing the state dynamics. This topic will come back in Case Study I of Section 3.1.

Secondly, all models more or less explicitly attempt to evolve solutions that mimic how humans solve this task, i.e., by perpetually crossing each other in an oscillatory pattern. This is often done by adopting implementation choices that are not cognitively plausible, in particular by introducing an artificial *sensory delay* initially proposed by Rohde and Di Paolo (2006) and preserved by others (Rohde and Di Paolo, 2006; Iizuka and Di Paolo, 2007; Di Paolo et al., 2008; Froese and Di Paolo, 2008; Froese and Di Paolo, 2010; Froese and Di Paolo, 2011). This choice does not seem well-justified since the work of Iizuka et al. (2015) suggests that sensory delay in human participants does not help the subjects to reach coordinated behavior. Izquierdo et al. (2022) is the only work that tries to move away from earlier assumptions and reports under which circumstances agents reproduce human-like behavior. In particular, contrary to what has been previously reported, they show that sensory delay is not necessary to evolve perpetual crossers. This is one of the implementation details which calls for a better way to make results replicable.

The current work aims at being neutral with respect to the type of agents' behavior that emerges from their interaction, since we believe it is important to explore and report on all possible solutions that are found.

## 2. PCE simulation toolkit

We developed a Python implementation of simulation-based PCE. The code allows for a wide range of experimental settings to be changed, such as the environment length, number of trials, the objective function, as well as the network architecture of the agents (i.e., number of neural nodes, and parameters range).

### 2.1. Simulation setup

What follows is the description of the general simulation setup which is common to every experimental setting.

Each simulation consists of a *number of trials*. In each trial, two agents are placed at random positions within the environment together with their shadows^4^ and the fixed objects. Agents interact for a specific *number of steps*, and their performance is assessed for a chosen *objective function* (such as the average distance between the agents). Finally, after all trials are over, the overall performance is computed based on some chosen *aggregate function* (such as mean or min) on the list of trial performances.

### 2.2. Agents network

**Figure 3:**
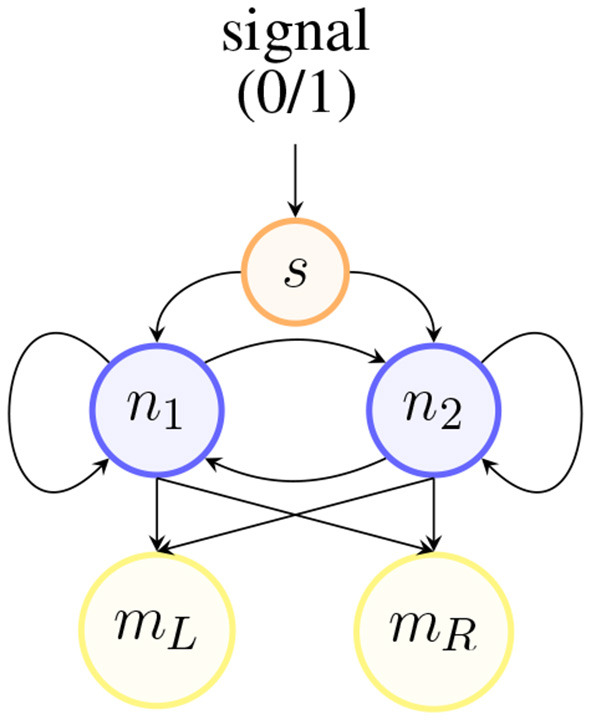
Example of a 2-neuron agent's network implementation.

The network architecture governing the behavior of each agent is shown in Figure 3. It consists of three layers: the sensory layer (top), the neural layer (middle), and the motor layer (bottom). All sensory nodes have connections toward all neurons; all neural nodes are fully connected: to all other neurons (in both directions) and to themselves; finally, both motor nodes receive input connections from all neural nodes. This architecture is the most simple and generic since it does not define any hierarchical structure within the neural layer.

The sensor *s*, at a specific time step, receives a binary signal *I*_*s*_∈{0, 1} in input. Its output $O_{s}^{S}$ is defined as:


(1) $$O_{s}^{S}=G_{s}\;\sigma (I_{s}+\theta_{s})$$

where *G*_*s*_ and θ_*s*_ are the sensory gain and bias, while σ is the standard activation sigmoid function 1/(1+*e*^−*x*^). Gains and biases will also be employed for neural nodes and motors. The first is used to alter the output range, whereas the bias allows for shifting the value of the input signals.

The sensor's output is propagated to each neural node *n*_*i*_ in layerN (depicted in blue in Figure 3), which can consist of any number of nodes. The nodes in this layer are implemented via a continuous-time recurrent neural network (CTRNN) as described by Beer (1995). We adopted this type of neural network because it can be easily integrated with evolutionary algorithms to evolve the network's parameters, and it is capable of modeling complex dynamical systems with multiple time-varying variables.

The delta of the neuron state Δ*y*_*n*_ between two consecutive time steps is calculated via the Euler method integration of the differential equation governing the state change, as follows:


(2) $$\Delta y_{n_{i}}=\frac{\Delta t}{\tau_{n_{i}}}\left(-y_{n_{i}}+W_{s,n_{i}}O_{s}^{S}+\sum_{j=1}^{\left|N\right|}W_{n_{j},n_{i}}O_{n_{j}}^{N}\right)$$

Here, τ_*n*_ is the neural time constant, Δ*t* is the steps size constant for the integration, and $O_{n}^{N}$ is the output of neuron *n*∈N calculated as:


(3) $$\begin{array}{l} O_{n}^{N}=G_{n}\;\sigma \left(y_{n}+\theta_{n}\right) \end{array}$$

with *G*_*n*_ and θ_*n*_ being the neural gain and bias.

Next, the output of each motor *m*∈M is calculated as:


(4) $$\begin{array}{l} O_{m}^{{}^{M}}=G_{m}\;\sigma \left(\overset{\left|N\right|}{\underset{n=1}{\sum }}W_{n,m}\;O_{n}^{N}+\theta_{m}\right) \end{array}$$

where *G*_*m*_ and θ_*m*_ are the motor gain and bias.

Finally, the displacement of the agent Δ*x* at every step is computed as the difference between the two motors:


(5) $$\begin{array}{l} \Delta x=O_{R}^{{}^{M}}-O_{L}^{{}^{M}} \end{array}$$

### 2.3. Evolutionary algorithm

As done in previous simulation studies, all network parameters are evolved for a given *number of generations*, using a genetic algorithm. The choice of adopting an evolutionary approach is justified by the fact that it is hard to formulate analytical solutions to this task and because it is the most effective way to explore a variety of solutions without introducing human biases. Each agent genotype is represented by an array of real-values in the fixed range [−1, 1]. At each generation, the parameters encoded in each agent's evolved genotype are converted to the correct ranges via linear interpolation.

For the evolutionary part, our implementation makes use of the *pyevolver*^5^ library which we previously introduced in Reséndiz-Benhumea et al. (2021). The library contains a Python implementation based on available C++ open-source code by Beer (1995) and offers many parameters to fine-tune the type of evolutionary process under study (such as mutation variance, and cross-over probability). In addition, it accommodates the possibility of evolving multiple populations of agents, which is relevant to using pairs of independent (non-clone) agents, such as in the second case study of the following section.

### 2.4. Parameter exploration

It is beyond the scope of this paper to provide a complete report on the simulation results using all possible combinations of parameters. However, since we have explored a fairly large number of combinations, we would like to provide some general observations on how a few important parameters affect the simulations.

a) *Objective Function:*
There are many ways in which agents can be rewarded for staying together as long as possible. Consistently with previous work, we implemented a *distance*-based function (described later in Equation 6). We also experimented with an *overlapping*-based function that measures the number of time steps in which the agents overlap each other. It seems that both functions are able to push the agents to find each other, but the *distance* function is more lenient to allow agents to stay close while not overlapping for some part of the interaction.
b) *Aggregate function:*
Most of previous work use the *mean* as the aggregate function of the performances of all trials. Consistently to what has been reported in Izquierdo et al. (2022), we noticed that this could easily lead to results with a low performance on a very small set of trials (which is not affecting too much the average). As an alternative strategy, we have also explored the use of the *min* as the aggregation function, so that the worst performance among all trials is taken as the final performance. This choice helps reach a higher number of successful simulations but might suffer from overfitting, especially if the number of trials tested is low.
c) *Object positioning:*
All previous models assume that static objects are placed at a fixed position across all trials (typically at opposite sides of the ring). We observed that agents evolved with such a positioning do not generalize to trials in which objects are disposed in a different manner. Therefore, we decided to mainly focus on the random disposition of objects. Even if these cases are harder to evolve, the ones that succeed result in a more robust solution.

## 3. Case studies

In this section, we illustrate two case studies where we vary the number of populations: in the first, one population is used, whereas, in the second one, two populations are used. In both cases, we test networks with a variable number of neural nodes: we start with 1 node as the most simple architecture and increase them up to 4. A higher number of nodes is likely to exhibit more complex behavior, which might be interesting as a follow-up study.

In both cases, we use the following experimental setup:

a) *Simulation:* as the *objective function*, we use the normalized average distance between two agents, defined as:

(6) $$\begin{array}{l} f=1-\frac{2\bar{d}}{L} \end{array}$$

where $\bar{d}$ is the average distance between the center points of the two agents within a trial, and *L* is the environment length. This function ranges between 0 (when agents always stay perfectly aligned) and 1 (when agents always stay at the opposite sides of the environment). As the *aggregate function* we use *min*, so that the worse performance among all the trials is used as the fitness value for the evolutionary algorithm. Moreover, we use the following parameters: environment length: 300 units; agents and objects length: 4 units; number of steps: 2, 000; number of trials: 30; agents positioning: random uniform for each trial; objects positioning: random uniform for each trial; shadow displacement^6^ from agent: 75 units;
b) *Network:* number of neurons: variable, from 1 to 4; gains range (*G*_*s*_, *G*_*m*_): [1, 10]; neural gain (*G*_*n*_): 1; biases range (θ_*s*_, θ_*n*_, θ_*m*_): [−3, 3]; weights range (*W*_*s*_): [−8, 8]; time constant range (τ): [1, 2]; steps size constant for the integration (Δ*t*): 0.1.
c) *Evolutionary Algorithm:* number of populations: 1 (case study I) and 2 (case study II); population size: 48; number of generations: 2, 000; fitness normalization mode: fitness proportionate selection; selection mode: roulette wheel selection; reproduction mode: genetic algorithm; mutation variance: 0.1; elitist fraction: 0.05; mating fraction: 0.85; filling fraction: 0.1; crossover probability: 0.5; crossover mode: uniform.

### 3.1. Case study I

In this first case study, we evolve a single population of agents, where each agent is tested against a *clone* of itself. This is consistent with what has been done in all previous models, except for Iizuka and Di Paolo (2007).

We evolved simulations for agents with 1, 2, 3, and 4 neurons, each with 40 random seed initializations. The random seed affected only the evolutionary algorithm in order to generate different evolved populations (all initial configurations of the trials were identical for all seeds). We observe that no solution was reached for agents with 1 and 2 neurons. To be precise, some solutions were found for the training settings (initial configurations of the trials during evolution), but none were robust to other initializations.^7^

**Figure 4:**
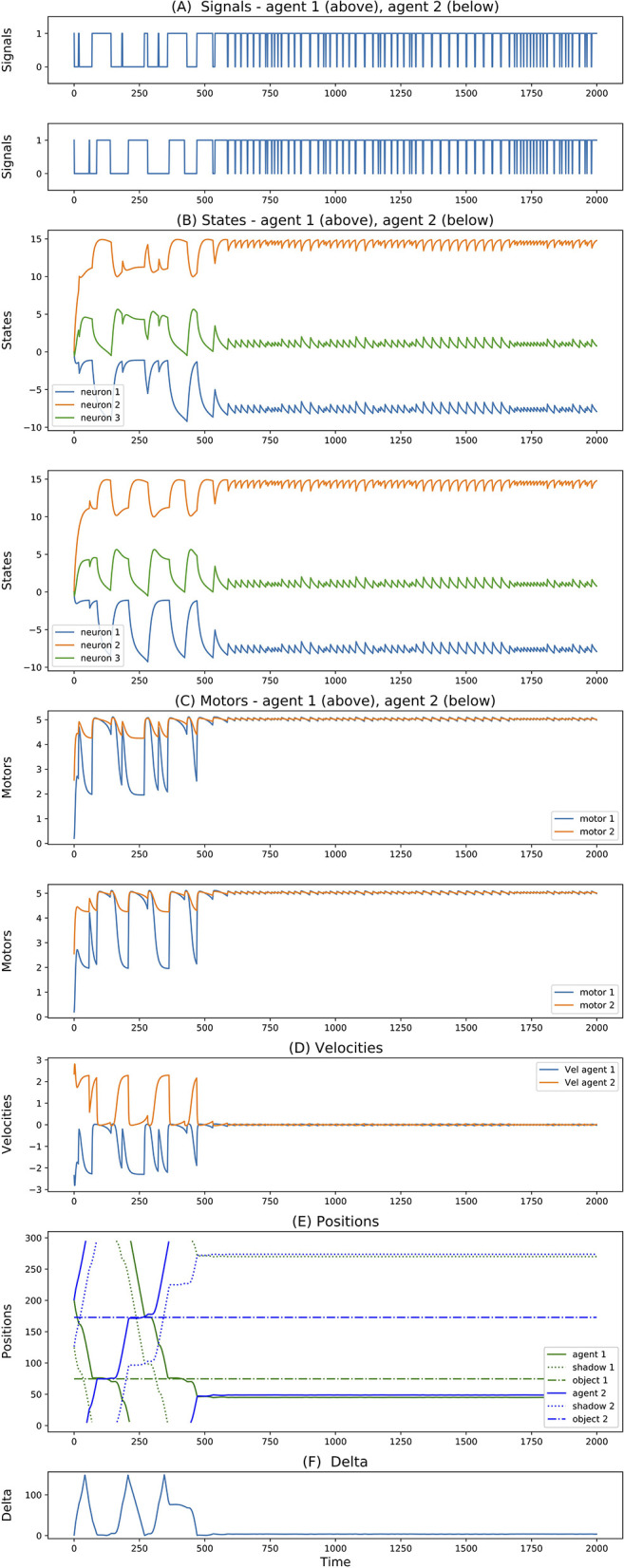
**(A–F)** Signals, states, motors, velocities, positions, delta of two best evolved agents of Case Study I (neurons = 3, seed = 24, trial = 23).

For simulations with 3-neuron agents, only 2 seeds reached successful and robust solutions. Both of them show very similar dynamics. One of them is reported in Figure 4. Here, we can see that both agents move in opposite directions. To avoid ambiguity, we always report the directions with respect to an external observer, but we should not forget that since agents are facing each other, this means that they are moving toward the same side (right-wards in this case) relative to themselves. When they meet (around step 500), they cross each other and repeat oscillating back and forward touching their edges perpetually. Most interesting, we can see that from this point onward, their state dynamics is identical and perfectly synchronized. We believe this is related to the agents dynamics previously reported for the 3-neuron manually-coded solution in Froese and Di Paolo (2010).

**Figure 5:**
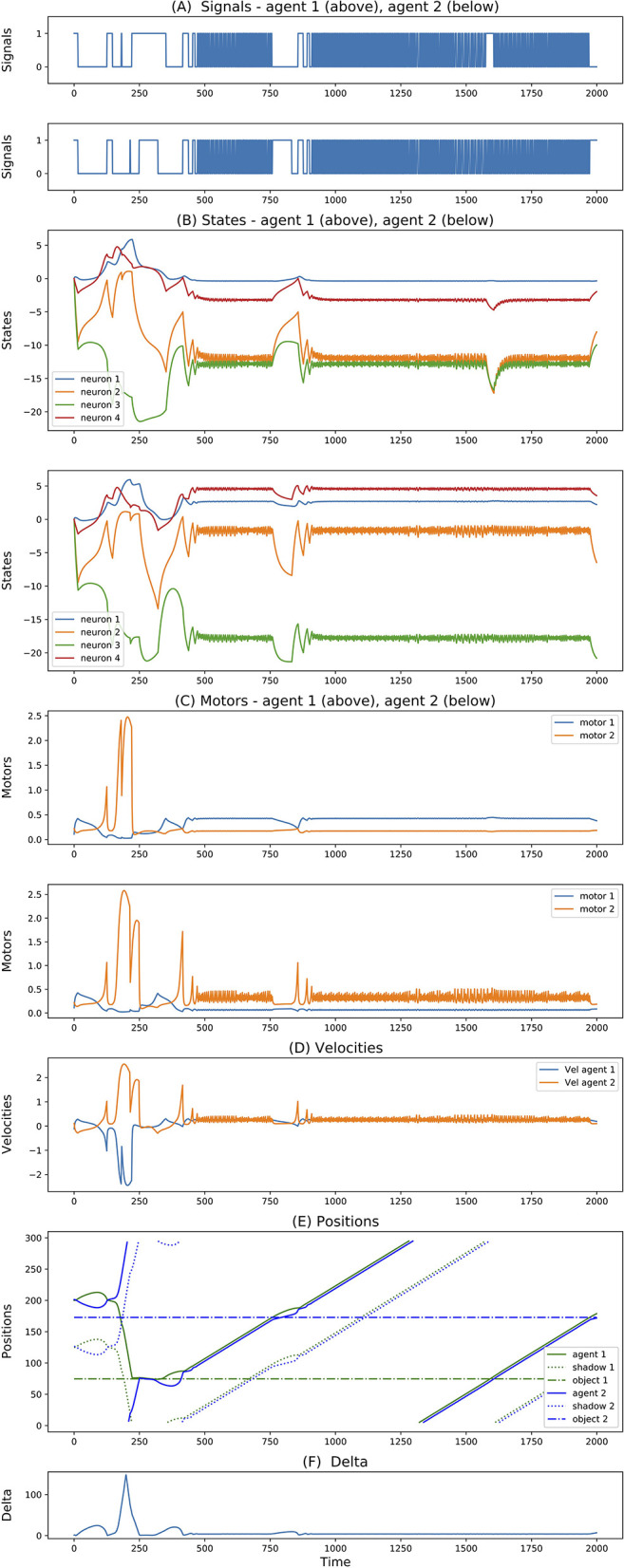
**(A–F)** Signals, states, motors, velocities, positions, delta of two best evolved agents of Case Study I (neurons = 4, seed = 23, trial = 23).

Finally, for simulations with 4-neuron agents, 3 seeds reached successful and robust solutions. While 2 of them present solutions similar to the one described above, the third one shows a rather different dynamics. This is illustrated in Figure 5. The difference here is that initially the agents both move in opposite directions (both left-wards with respect to themselves). After crossing each other a number of times and both inverting their directions, around step 400 they end up crossing each other and moving in the same direction. More interestingly, when looking at the internal states, while before step 400 the state dynamics is rather similar between the two agents, after this point it follows completely different trajectories. This outcome seems related to what was reported in experiment 3 of Froese and Di Paolo (2011) (even though in that case they explicitly reward agents for traveling together, and there is no report of their states dynamics).

### 3.2. Case study II

In the second case study, we evolve two populations of agents so that the pair of agents interacting in the simulation are evolved independently. However, the agents interacting in the simulation are those sharing the same rank in the two populations (i.e., the n-th best agent of the first population is paired with the n-th best agent of the second population). Since the objective function (normalized distance) is common for both agents, each pair stays aligned across the various generations, even though the individual agent may undergo separate mutation processes.

We evolved simulations for agents with 1, 2, 3, and 4 neurons, each with 10 random seed initializations. The random seed affected only the evolutionary algorithm in order to generate different evolved populations. Differently from the previous case study, all seeds resulted in successful solutions (min performance among all trials was greater than 0.9) including for agents with a single neuron. We also tested the robustness of the solution for different random initialization of the positions of the agents and fixed objects and varying the distance of the shadows, and confirmed that all solutions are robust to those changes.

**Figure 6:**
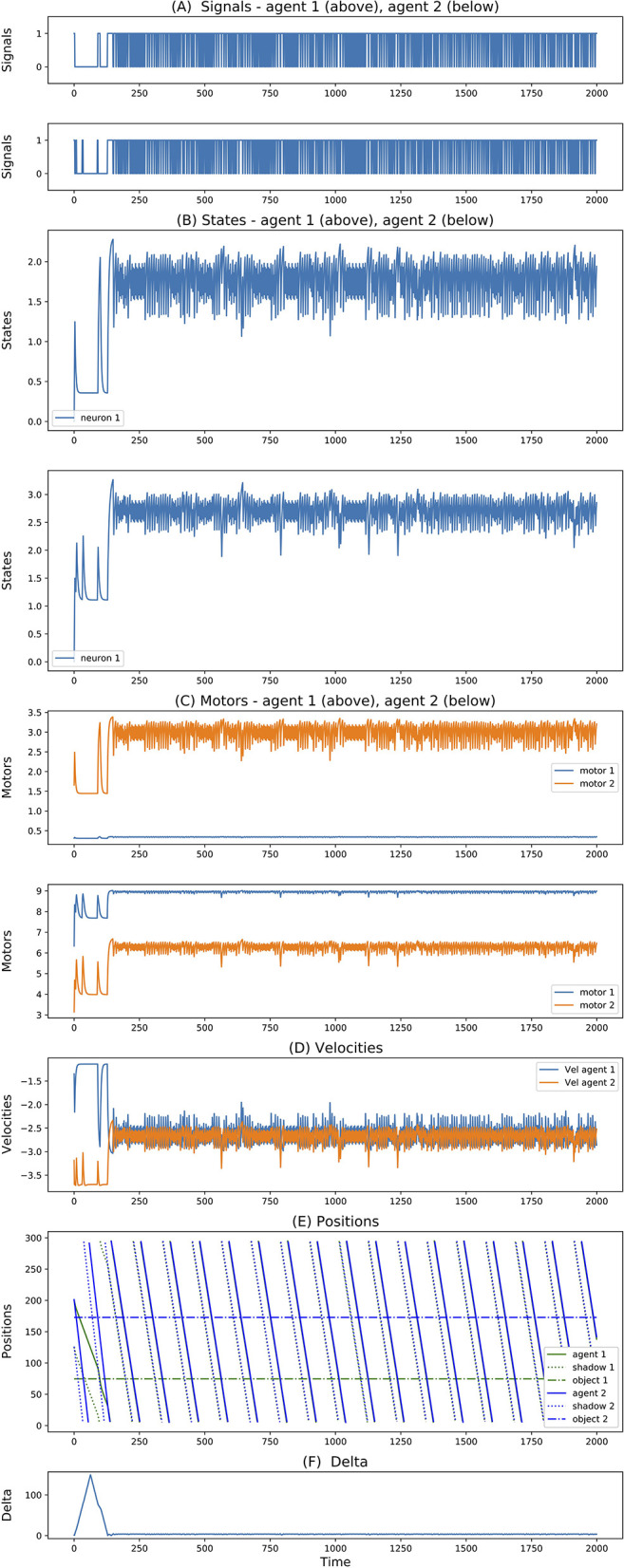
**(A–F)** Signals, states, motors, velocities, positions, delta of two best evolved agents in Case Study II (neurons = 1, seed = 3, trial = 23).

**Figure 7:**
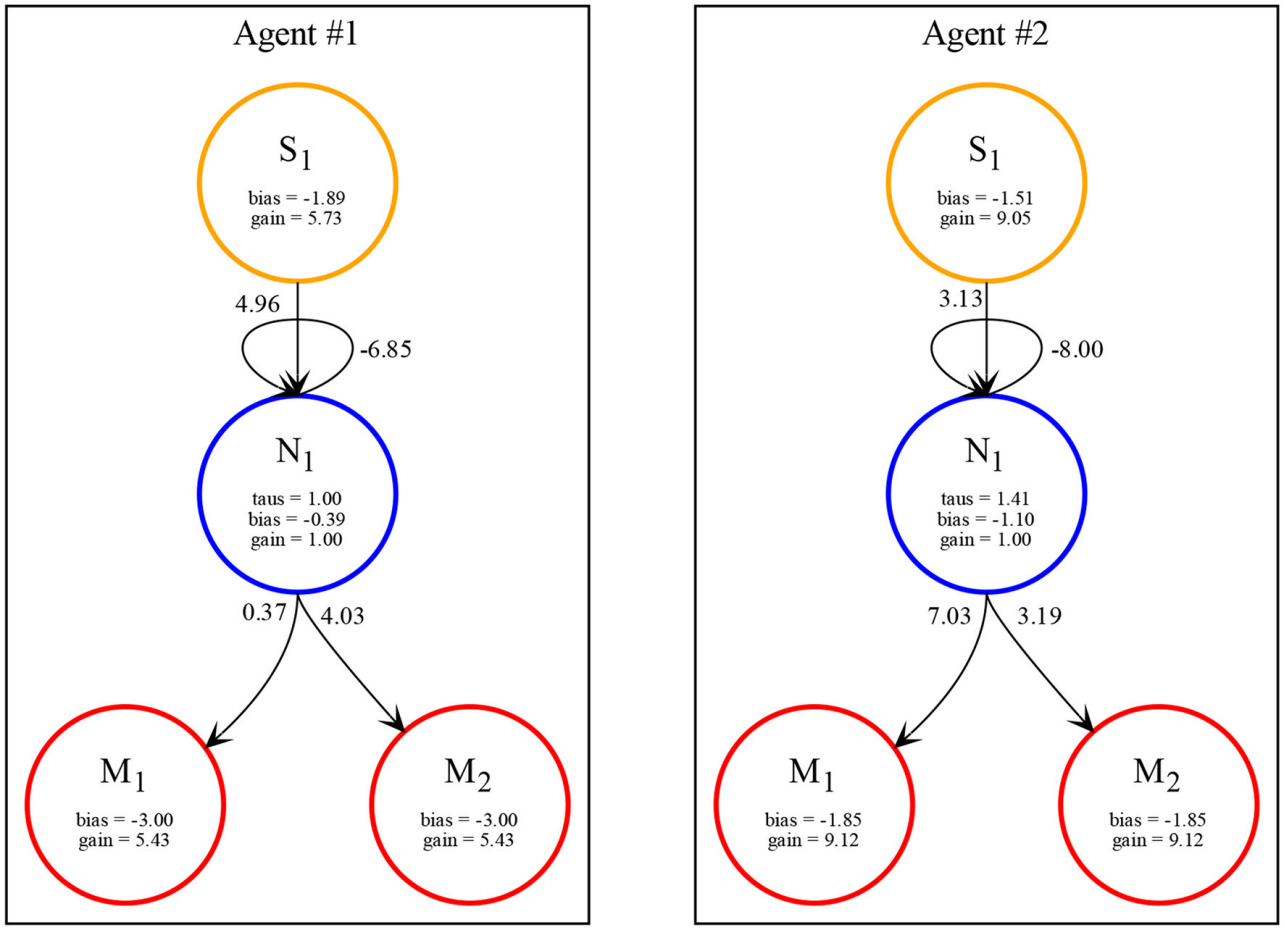
Network with parameters of the two best evolved agents in Case Study II (neurons = 1, seed = 3, trial = 23) automatically rendered by our toolkit.

In all the simulations, we observe that agents exploit complementary strategies to find each other. A typical behavior is illustrated in Figure 6. Here, both agents move clockwise from the perspective of an external observer (although they move in opposite directions with respect to themselves because they are facing opposite sides of the environment). However, while the first agent goes slow and accelerates only when perceiving any other object, the second agent goes fast and decelerates only when perceiving any other object. Because the two agents evolved together, they were able to fine-tune the final velocities of when they both cross each other to be about the same (around 2.5 units per step), but since the final velocity is not exactly the same, this leads to a repeated catch-and-wait oscillatory dynamics. The network and parameters of the two agents is illustrated in Figure 7.

## 4. Conclusions and future work

In this paper, we presented the first open-source toolkit for PCE simulations. We hope this framework will stimulate more replicable results in this type of research in the future. We described the various parameters that it offers, and demonstrated how it can evolve successful solutions in two case studies. It is important to stress that our implementation did not include any artificial sensory delay, and consistently to Izquierdo et al. (2022) (but contrary to previous work), we confirm it is not necessary to evolve successful solutions. In the first case study, we detailed some specific solution evolved for agent clones (single population) consistent with previous literature. In the second case, we reported on a very simple solution evolved by pairs of single neuron agents exhibiting complementary strategies. The triviality of the solution reflects a number of simple assumptions underlying the experiments that we wish to remove in future work: first, we would like to explore what complementary strategies could arise in a simulation where *noise* is introduced (both in the signals and in the motors), similar to what has been done in Froese and Di Paolo (2008). Second, we would like to bring the experimental setup closer to the human-subject experiments where each agent is required to activate an additional motor to press a button when it is confident to be crossing the other agent. Finally, a completely different scenario to be explored, which is currently supported by the toolkit, is to generalize the PCE simulation to more than 2 agents.

From a user perspective, the toolkit would benefit from implementing additional functionalities to simplify parameter exploration and analysis of resulting solutions. We aim to continue this work, but we will welcome the contributions of other researchers to this open project.

## Data availability statement

The dataset and code of the toolkit presented in this paper can be found in the online repository at: https://github.com/oist/ecsu-cnru-pce-simulation.

## Author contributions

FS conceived the idea for this paper, developed the code of the PCE Simulation Toolkit, and wrote the manuscript. RF verified the simulation results, supported the writing of the manuscript, and contributed to the development of the final plots. Both authors contributed to the article and approved the submitted version.

## Footnotes

^1^ https://github.com/oist/ecsu-cnru-pce-simulation

^2^As an indication, each experiment reported in the case studies at the end of this contribution took around 6 h on a 128 AMD Epyc CPUs node.

^3^The sensor outputs 1 when it crosses any part of an intervening object; otherwise, it outputs 0.

^4^In the current implementation, consistently to previous implementation, the shadows are always located at a constant distance from the respective agents.

^5^ https://github.com/oist/ecsu-pyevolver

^6^The displacement is calculated clockwise for both agents.

^7^These could be considered cases of overfitting, and we still need to test if a higher number of trials would prevent this from happening.
